# Closed-Loop Neuromodulation for Brain Fatigue: From Real-Time Biomarkers to Adaptive Intervention

**DOI:** 10.3390/ijms27177970

**Published:** 2026-09-07

**Authors:** Hongliang Lu, Yajuan Zhang, Shengjun Wu, Danmin Miao

**Affiliations:** Department of Military Medical Psychology, Air Force Medical University, Xi’an 710032, China; 18066872356@163.com (Y.Z.); psych@fmmu.edu.cn (D.M.)

**Keywords:** brain fatigue, closed-loop neuromodulation, molecular biomarkers, neuroinflammation, adaptive intervention

## Abstract

Brain fatigue is a debilitating condition whose molecular complexity—neurotransmitter imbalance, neuroinflammation, and metabolic failure—has long defied effective intervention. Conventional open-loop neuromodulation offers fixed stimulation, but fatigue fluctuates. Closed-loop neuromodulation, adjusting stimulation in real time, holds real promise. Yet its delivery hinges on a single, unresolved bottleneck: the sensing–decision chain. In this review, we deconstruct closed-loop systems into sensing, decision, and intervention, and argue that the true challenge is not technological but informational—how to integrate fast electrophysiological signals with slow molecular biomarkers (inflammatory cytokines, neurotrophic factors, adenosine) into a unified control framework. We show that a “fast-slow variable” architecture offers a practical path forward: fast signals guide immediate responses, slow variables set baselines and thresholds, and their integration enables predictive, pre-emptive intervention. We also examine the molecular correlates of neuromodulation—synaptic plasticity, anti-inflammatory signaling, and neurotrophic regulation—as the mechanistic foundation for therapeutic effect. Finally, we confront the translational triad of causality, inter-individual variability, and the information–energy–time trade-off. We conclude that realizing effective closed-loop neuromodulation for brain fatigue will require parallel advances in both stimulation hardware—improving spatial targeting, dose precision, and modality versatility—and individualized sensing–decision algorithms capable of reliably translating multi-modal biomarkers into timely, safe interventions. Both areas remain active fronts of development, and neither can be neglected in clinical translation.

## 1. Introduction

Brain fatigue is defined here as a subjective state of persistent mental exhaustion that is not relieved by rest and is accompanied by measurable declines in cognitive performance or neural function [1]. It is increasingly recognised as a major public health concern that transcends diagnostic boundaries, with debilitating effects across neurological, psychiatric, and systemic autoimmune disorders [2,3,4]. Furthermore, it is a core feature of ME/CFS and Long COVID, where it profoundly diminishes quality of life [5,6]. The clinical and societal burden is substantial, manifesting as impaired cognitive performance, reduced workplace productivity, and diminished well-being, yet it remains poorly managed due to incomplete understanding of its mechanisms and a lack of effective adaptive interventions [7,8,9,10,11,12]. Clinically, it presents as persistent exhaustion disproportionate to activity, effortfulness during routine tasks, and non-restorative rest, often with reduced attention, slowed processing, and emotional lability—distinct from major depressive disorder, early Alzheimer’s disease, and primary sleep disorders [13].

At the molecular level, brain fatigue is understood as a complex state arising from the interplay of multiple biological pathways [12,14]. Dysregulation of neurotransmitter systems, particularly dopamine which is crucial for motivation and reward, is a key factor [15]. Concurrently, neuroinflammation, driven by pro-inflammatory cytokines, has been consistently implicated in fatigue across diverse conditions [16,17,18,19]. These inflammatory signals can disrupt central nervous system (CNS) homeostasis and contribute to the persistent feeling of exhaustion [20]. Metabolic dysfunction, including impaired ATP generation and mitochondrial dysfunction, further compounds the pathology [21]. Lactate, once considered a mere metabolic waste product, is now recognised as a key regulator in the CNS, linking metabolic state to neuronal function and potentially neuroprotection [22]. These molecular alterations form an interacting network, creating a self-sustaining pathological state that underscores why a single biomarker or fixed intervention is unlikely to be sufficient [23,24].

The molecular complexity of brain fatigue is perhaps best illustrated by the reciprocal relationships among its three core pathological domains. Neuroinflammation, driven by elevated pro-inflammatory cytokines, has been shown to directly affect neurotransmitter metabolism through the indoleamine-2,3-dioxygenase (IDO) pathway [25,26], which shifts tryptophan metabolism away from serotonin synthesis and toward the production of neurotoxic kynurenine metabolites [25,26,27]. This metabolic diversion simultaneously reduces serotonin availability—contributing to the motivational and affective components of fatigue—and generates quinolinic acid, an NMDA receptor agonist that can promote excitotoxicity and further neuroinflammatory activation [28,29]. Meanwhile, the energetic demands of sustained neuroinflammation exacerbate mitochondrial dysfunction and ATP depletion [30], while adenosine accumulation from ATP breakdown inhibits dopaminergic transmission and promotes sleep-promoting neuronal activity. This three-way interaction—inflammation impairing neurotransmitter function and energy metabolism, metabolic dysfunction sustaining inflammation through oxidative stress [31,32], and neurotransmitter imbalance reducing the motivation to engage in restorative behaviors—creates a self-perpetuating cycle that explains both the persistence and the dynamic fluctuation of brain fatigue. This complex systems perspective has profound implications for intervention design [33,34]: it suggests that targeting any single pathway in isolation, whether pharmacologically or through fixed neuromodulation protocols, is unlikely to produce durable therapeutic effects unless the system as a whole can be steered toward a more adaptive equilibrium [35,36].

Conventional neuromodulation approaches for brain fatigue have predominantly operated in an open-loop manner, delivering stimulation with fixed parameters based on predetermined protocols [37]. While such approaches have shown some efficacy in controlled research settings—for instance, standard tDCS protocols applied to the dorsolateral prefrontal cortex have demonstrated modest but inconsistent effects on fatigue reduction in multiple sclerosis patients—their outcomes are often highly variable across individuals and across studies [38,39,40]. A key reason for this inconsistency is that open-loop systems cannot accommodate the dynamic nature of fatigue, which fluctuates over timescales ranging from minutes to days, nor can they adjust to individual differences in brain anatomy [41], baseline excitability, or the specific molecular drivers of fatigue in a given person. A fixed-parameter stimulation protocol delivered at a fixed time of day, regardless of the brain’s current state or the individual’s fluctuating fatigue level, is inherently mismatched to the phenomenon it seeks to treat [42,43]. For example, the same anodal tDCS protocol that effectively reduces fatigue in one patient may have no effect or even worsen fatigue in another, depending on the individual’s baseline cortical excitability, the phase of their circadian rhythm, and the underlying neuroinflammatory status—variables that are ignored by conventional open-loop approaches [44,45].

Closed-loop neuromodulation offers a paradigmatic alternative [46,47,48]. By integrating real-time physiological sensing with adaptive decision-making and targeted intervention, closed-loop systems can theoretically adjust stimulation parameters in response to the brain’s fluctuating state, delivering the right intervention at the right time [49,50]. This architecture, comprising sensing, decision-making, and intervention modules, has already shown promise in other neurological disorders including epilepsy, where responsive neurostimulation systems detect seizure onsets and abort them before clinical manifestations [51,52], and Parkinson’s disease, where adaptive deep brain stimulation adjusts stimulation amplitude in real time based on subthalamic local field potential biomarkers [53]. The successful translation of closed-loop principles to these conditions demonstrates the feasibility of the approach and provides a template for its application to brain fatigue [54,55,56]. However, the extension to fatigue presents unique challenges that are not encountered in epilepsy or Parkinson’s disease: fatigue lacks a clear “ictal” event, its biomarkers are more subtle and context-dependent [57,58,59], and the optimal intervention may vary not only across individuals but also within the same individual from day to day [60,61].

This review is structured as a critical narrative review, aiming to evaluate the current evidence and conceptual framework for closed-loop neuromodulation in brain fatigue, with a focus on integrating electrophysiological and molecular biomarkers into the sensing–decision–intervention architecture. Our literature coverage draws from PubMed, Web of Science, and Scopus (2000–2026), prioritizing peer-reviewed original articles, systematic reviews, and meta-analyses, with preclinical studies included only for mechanistic insights not available from human data. The primary scope encompasses clinical fatigue conditions—ME/CFS, Long COVID, multiple sclerosis, cancer-related fatigue, and systemic inflammatory disease-associated fatigue—while occupational and cognitive fatigue are discussed primarily for biomarker validation. Our central thesis is that the primary bottleneck is not stimulation technology but the reliability and individualization of the sensing–decision chain. We proceed from the molecular pathophysiology of brain fatigue, through real-time biomarkers and triggering logics, to adaptive intervention strategies and their translational challenges, concluding with future directions and a synthesis of our core arguments.

## 2. The Molecular Pathophysiology of Brain Fatigue

Understanding the molecular pathophysiology of brain fatigue is a prerequisite for designing rational closed-loop systems. Without a clear picture of the molecular events that underlie fatigue initiation and persistence, the selection of sensing targets and intervention strategies remains largely empirical. However, brain fatigue is not a single entity—its presentation, time course, and biological underpinnings differ substantially across clinical contexts. Table 1 provides a phenotypic classification that distinguishes these subtypes and their respective implications for closed-loop system design. This section synthesizes current evidence on three interconnected molecular domains: neurotransmitter dysregulation, neuroinflammation, and metabolic dysfunction, which collectively drive and sustain the fatigue state through a complex network of reciprocal interactions.

### 2.1. Neurotransmitter Dysregulation

The monoamine hypothesis of central fatigue posits that an imbalance in brain monoamine neurotransmitters—particularly dopamine, serotonin, and norepinephrine—plays a causal role in the development of fatigue [62]. Among these, dopamine has received the most attention for its role in motivation, reward processing, and the willingness to exert effort—the very behavioral dimensions that are compromised in fatigue states [15,63,64]. A reduction in dopaminergic tone in the basal ganglia and prefrontal cortex reduces the perceived value of effort and the motivation to engage in cognitively demanding tasks [65], contributing directly to the behavioral manifestations of fatigue that are often described as “apathy” or “lack of drive.” Animal models have provided causal evidence for this pathway: when the cytokine TGF-β was experimentally elevated in the brain, it suppressed dopamine production and induced fatigue-like behaviors [66,67], establishing a direct molecular link between inflammatory signaling, dopaminergic function, and fatigue.

Serotonin, in contrast to dopamine, is thought to influence fatigue through its effects on motor output and the brain’s effort–reward evaluation system. Elevated serotonergic activity, particularly in the basal ganglia and anterior cingulate cortex, has been consistently associated with increased fatigue perception during sustained physical or cognitive exertion [68]. The mechanism involves serotonin’s inhibitory effects on motor neuron pools and its modulation of the subjective cost of effort, making tasks feel more effortful and less rewarding [69,70]. The interaction between dopamine and serotonin systems is particularly important: serotonergic and dopaminergic pathways have antagonistic effects on effort–reward trade-offs [71], and the balance between these two systems may be more relevant to fatigue than the absolute level of either transmitter alone [72,73]. This is exemplified by the observation that interventions that increase dopamine while decreasing serotonin—such as certain pharmacological and neuromodulatory approaches—tend to produce the most robust anti-fatigue effects.

Norepinephrine, which regulates arousal, vigilance, and attention through its projections from the locus coeruleus to widespread cortical and subcortical regions, also shows altered dynamics in fatigue states [74]. Reduced noradrenergic tone impairs sustained attention and increases distractibility, compounding the cognitive impairments associated with fatigue [75]. Furthermore, the excitatory–inhibitory balance maintained by glutamate and GABA is disrupted in fatigue [75], with evidence pointing to elevated glutamate levels in the anterior cingulate cortex and insula—regions that are central to interoceptive processing [76,77], effort evaluation, and the subjective experience of exhaustion [76]. This imbalance may arise from impaired glutamate clearance by astrocytes under conditions of metabolic stress or neuroinflammation, leading to excitotoxic effects that further compromise neuronal function and synaptic transmission [78,79]. Collectively, these neurotransmitter alterations do not act independently; they interact in complex ways [80]—serotonergic–dopaminergic antagonism, noradrenergic modulation of dopamine release, and glutamatergic influence on GABAergic inhibition—forming a network that sustains the fatigued state [81].

### 2.2. Neuroinflammation and Cytokine Pathways

Neuroinflammation has emerged as a key driver of brain fatigue across diverse clinical conditions [82,83]. In illnesses like ME/CFS, a shift from a transient to a chronic immune/inflammatory response is thought to be a central mechanism [84], leading to activation of the brain’s specific immune system and resulting neuroinflammation [85,86]. Pro-inflammatory cytokines—particularly interleukin-6 (IL-6), tumor necrosis factor-alpha (TNF-α), and interleukin-1 beta—have been repeatedly observed at elevated levels in the peripheral blood of individuals with fatigue-predominant disorders [87], with some evidence of CSF elevation as well. These peripheral signals can access the central nervous system through multiple routes: active transport across the blood–brain barrier via saturable transport systems [88,89], binding to receptors on the cerebral vasculature that trigger local inflammatory signaling, or entry through circumventricular organs where the blood–brain barrier is inherently leaky [90].

Once in the brain, these cytokines activate microglia and astrocytes through both canonical NF-κB signaling and non-canonical pathways, triggering a cascade of central inflammatory responses. Microglial activation has been documented in fatigue conditions using translocator protein (TSPO) positron emission tomography (PET), with increased TSPO binding observed in regions including the anterior cingulate cortex, insula, and thalamus—regions that overlap substantially with the brain’s interoceptive and salience networks [91,92,93]. Importantly, microglial activation is not merely a bystander phenomenon [85,94]; activated microglia release their own pro-inflammatory cytokines, creating a self-amplifying loop that can sustain neuroinflammation even after the initial peripheral trigger has resolved [95]. This self-sustaining nature of neuroinflammation may explain the persistence of fatigue long after the resolution of the inciting infection or injury in conditions like post-COVID syndrome [96].

The molecular link between neuroinflammation and neurotransmitter systems is perhaps the most critical intersection for understanding fatigue [97,98]. Inflammatory cytokines affect serotonin metabolism through the IDO pathway: IDO converts tryptophan to kynurenine, reducing the availability of tryptophan for serotonin synthesis and generating kynurenine metabolites, including quinolinic acid, which have neurotoxic and pro-inflammatory effects [99]. Similarly, inflammatory cytokines affect dopamine metabolism by reducing the availability of tetrahydrobiopterin (BH4), a critical cofactor for tyrosine hydroxylase, the rate-limiting enzyme in dopamine synthesis [100]. These convergent effects on both serotonin and dopamine systems provide a mechanistic explanation for the co-occurrence of fatigue with mood disturbances, cognitive impairment, and reduced motivation—the “sickness behavior” complex that is evolutionarily conserved and reflects a fundamental reallocation of metabolic resources toward immune defense. The IDO–kynurenine pathway is particularly relevant to closed-loop system design because kynurenine metabolites have been measured in cerebrospinal fluid and show correlations with fatigue severity that may be more robust than circulating cytokine levels alone [101,102,103,104].

### 2.3. Metabolic Dysregulation and Energy Failure

At the most fundamental level, brain fatigue reflects a state of cellular energy crisis. The brain is an energy-intensive organ, consuming approximately 20% of the body’s oxygen despite constituting only 2% of body mass [105], and it has limited capacity for energy storage, making it highly dependent on continuous glucose and oxygen delivery [106]. Sustained cognitive or physical demand places substantial metabolic demands on neural tissue, and when these demands exceed the brain’s metabolic capacity, a cascade of molecular events ensues that culminates in the subjective experience of fatigue.

ATP depletion is the central event in this cascade [107,108]. As ATP is consumed faster than it can be regenerated through oxidative phosphorylation, adenosine accumulates through the breakdown of ADP and AMP. Adenosine is a potent neuromodulator that promotes sleep and inhibits arousal [109,110]; it acts on A1 receptors to reduce neuronal firing and neurotransmitter release, and on A2A receptors in the basal forebrain to promote sleep onset [110]. Adenosine accumulation in the basal forebrain and prefrontal cortex promotes theta and delta EEG activity, directly contributing to the subjective experience of drowsiness and reduced alertness—a mechanism that is exploited by caffeine, which blocks adenosine receptors [111,112]. Importantly, adenosine accumulation also inhibits dopaminergic transmission through interactions between A2A and dopamine D2 receptors, providing a direct molecular link between energy failure and the motivational deficits of fatigue [113].

Mitochondrial dysfunction, oxidative stress, and impaired glucose metabolism have all been documented in fatigue-predominant conditions [114,115]. Studies examining cerebrospinal fluid in ME/CFS patients after exercise have provided objective evidence of metabolic dysfunction, showing altered consumption of lipids and metabolites compared to healthy controls [116]. Mitochondrial dysfunction may be both a cause and a consequence of neuroinflammation: inflammatory cytokines inhibit mitochondrial respiration and promote the production of reactive oxygen species [117], while mitochondrial damage and oxidative stress trigger inflammatory signaling through danger-associated molecular patterns (DAMPs) and activation of the NLRP3 inflammasome [118,119]. This reciprocal relationship between energy failure and neuroinflammation creates a vicious cycle that sustains both processes [120]. A particularly exciting recent development is the discovery of protein lactylation as a metabolic–epigenetic mechanism linking energy state to gene expression [121]. Lactate accumulation, long considered merely a metabolic waste product of anaerobic glycolysis, now appears to function as a signaling molecule [122,123]. Histone lactylation modifies chromatin structure and regulates the transcription of genes involved in inflammation and metabolism, providing a direct mechanism by which metabolic stress can shape inflammatory gene expression and sustain a chronic inflammatory state [124,125]. This finding suggests that the molecular pathophysiology of brain fatigue operates across multiple timescales, from rapid changes in neurotransmitter release and receptor binding, through intermediate changes in gene expression and protein synthesis, to long-lasting epigenetic modifications that may represent a “molecular memory” of the fatigue state.

### 2.4. Section Conclusion

The molecular pathophysiology of brain fatigue is characterized by the reciprocal interactions among neurotransmitter dysregulation, neuroinflammation, and metabolic dysfunction [75]. These three domains are not parallel pathways but rather intersecting and mutually reinforcing systems—each can trigger or worsen the others, creating a self-sustaining network that maintains the fatigue state even after the initial trigger has resolved. This complexity has direct implications for closed-loop system design: first, it suggests that effective sensing may require a combination of biomarkers reflecting different molecular domains [126]; second, it implies that intervention strategies should ideally target multiple pathways rather than a single molecular node [127]; and third, it underscores the importance of understanding the temporal dynamics of these molecular processes, which operate on different timescales—from seconds to hours to days—and may require adaptive algorithms that can accommodate this heterogeneity [128,129]. This molecular landscape provides the biological rationale for the biomarkers and interventions discussed in the following sections, and it suggests that the most effective closed-loop systems will be those that can sense and modulate across these multiple, interacting levels of biology. The key molecular mechanisms discussed above are summarized in Table 2, which also maps them to their potential roles as biomarkers and intervention targets in closed-loop systems.

**Table 2:** Core molecular pathophysiological mechanisms of brain fatigue and their potential roles as monitoring biomarkers or intervention targets.

Pathological Domain	Key Molecules/Pathways	Contribution to Fatigue	Molecular Effects of Neuromodulation	Potential Biomarkers	Temporal Scale	Evidence Level
Neurotransmitter Dysregulation	Dopamine (DA) ↓ Serotonin (5-HT) ↑ Norepinephrine (NE) ↓ Glutamate (Glu)/GABA imbalance	DA ↓: reduced motivation and effort willingness [15,63,64] 5-HT ↑: increased perceived effort cost [69,70] NE ↓: impaired sustained attention and increased distractibility [75,130] Glu/GABA imbalance: excitotoxicity risk [79]	tDCS/tACS: modulation of NMDA receptor-dependent LTP/LTD [131] Shifting Glu/GABA balance [132,133] CaMKII activation and AMPA receptor trafficking [134,135,136]	EEG spectral power (β/θ ratio, frontal alpha asymmetry) EEG functional connectivity (PLV, graph metrics)	Milliseconds–seconds (Real time)	★★
Neuroinflammation	Pro-inflammatory cytokines (IL-6, TNF-α, IL-1β) ↑ IDO–kynurenine pathway ↑ Microglial activation	Induction of sickness behavior [16,17,18,19] IDO pathway: 5-HT synthesis ↓, kynurenine ↑ [25,26,27] Microglial self-amplifying loop [85,86,95] BH4 depletion: DA synthesis ↓ [100]	taVNS: activation of cholinergic anti-inflammatory pathway [137,138] NF-κB inhibition, reduced pro-inflammatory cytokine release [138] Modulation of microglial activation state [44]	Inflammatory cytokines (IL-6, TNF-α, IL-1β) TSPO-PET (microglial activation) Kynurenine metabolites	Minutes–days (Slow)	★★
Metabolic Dysfunction	ATP depletion Adenosine ↑ Lactate ↑ Mitochondrial dysfunction	Cellular energy crisis [107,108] Adenosine ↑: sleep promotion, arousal inhibition [109,110] DA inhibition via A2A–D2 interaction [113] Histone lactylation: epigenetic regulation of inflammatory genes [121,124,125]	(Indirect) improvement of energy metabolism Modulation of adenosine signaling Regulation of lactate–lactylation axis Improvement of mitochondrial function (under investigation)	Lactate Adenosine (emerging) HRV (autonomic–metabolic coupling) NAA/MRS (neuronal integrity)	Seconds–days (Medium–slow)	★
HPA Axis Dysregulation	Cortisol ↓ CRH ↑	Chronic HPA axis suppression after prolonged stress [139] Disruption of energy regulation and sleep–wake cycles [140] Immune–neuroendocrine crosstalk	taVNS: modulation of HPA axis activity tDCS: potential hypothalamic modulation via limbic circuits	Salivary/serum cortisol (diurnal rhythm pattern) CRH	Minutes–days (Slow)	★
Neurotrophic Support	BDNF ↓ TrkB signaling ↓	Impaired synaptic plasticity [141,142] Reduced neuronal survival and LTP May mediate persistence and reversibility of fatigue	tDCS/tACS: BDNF upregulation [143,144] Activity-dependent BDNF secretion ↑ MAPK/ERK and PI3K/Akt pathway activation [145]	Serum/plasma BDNF BDNF Val66Met genotype	Hours–days (Slow)	★

Arrows indicate the typical direction of change in fatigue states (↑ increased; ↓ decreased). Temporal scales are categorized for different closed-loop applications: fast (real-time triggering), medium–slow (state tracking), and slow (baseline calibration and predictive algorithms). Evidence Level key: ★★ = strong correlational or mechanistic evidence, but closed-loop validation incomplete; ★ = preliminary or indirect evidence, currently exploratory. For neurotransmitter dysregulation markers (e.g., EEG spectral features), the ★★ rating reflects evidence primarily from occupational/cognitive fatigue in laboratory settings; generalization to clinical fatigue populations awaits further validation. It is important to distinguish between biomarkers suitable for real-time closed-loop control and those useful for long-term monitoring or baseline stratification. Only neurophysiological signals (EEG spectral features, connectivity metrics) provide the sub-second temporal resolution required for instantaneous stimulation adaptation. Molecular, inflammatory, metabolic, and HPA-axis markers operate on timescales of minutes to days and are therefore not suitable as real-time control signals; their role is best understood as baseline calibration, threshold adjustment, and outcome monitoring. This distinction is elaborated in Section 3 and Table 3. Abbreviations: LTP, long-term potentiation; LTD, long-term depression; PLV, phase-locking value; TSPO, translocator protein; NAA, N-acetylaspartate; MRS, magnetic resonance spectroscopy; CRH, corticotropin-releasing hormone; TrkB, tropomyosin receptor kinase B; BH4, tetrahydrobiopterin.

**Table 3:** Evidence readiness map for candidate biomarkers in closed-loop fatigue sensing.

Biomarker	Modality	Primary Confounders	Validation Status	Real-Time Feasibility	Recommended Role	Evidence Level
Beta/theta ratio	EEG (spectral)	Sleep deprivation, task difficulty, emotional arousal, caffeine	Validated in occupational/cognitive fatigue; limited in clinical fatigue	Yes (continuous)	Fast trigger (multimodal)	★★
Frontal alpha asymmetry	EEG (spectral)	Mood, motivation, individual baseline differences	Correlates with effort-based fatigue measures; limited replication	Yes (continuous)	Fast trigger (motivational component)	★
PLV/graph metrics	EEG (connectivity)	Reference electrode choice, artifact, head movement	Shown in fatigue studies; limited replication; high inter-individual variability	Yes (but computationally heavier)	Fast trigger (network integration)	★
HRV (RMSSD, LF/HF)	ECG/PPG	Physical activity, respiration, emotional arousal, medication, caffeine, age, fitness	Correlated with sustained task fatigue; confounders severe	Yes (but 5 min windows for frequency-domain metrics)	Slow baseline/contextual	★★
HEP	EEG + ECG	Interoceptive accuracy, anxiety, SNR	Preliminary in fatigue; very limited validation	Theoretically yes, but SNR poor	Research/exploratory	★
Peripheral cytokines (IL-6, TNF-α, IL-1β)	Blood/saliva/sweat	Circadian variation, infection, physical activity, BMI, medication	Consistently elevated in ME/CFS, Long COVID, cancer fatigue; correlational only	Not yet (biosensor development required)	Slow baseline calibration	★
Cortisol (diurnal pattern)	Saliva (multiple timepoints)	Sleep, stress, sampling time, season, medication	Reduced awakening response and flattened slope in ME/CFS, burnout	Not yet (multiple daily samples required)	Slow baseline/chronobiological adjustment	★
BDNF	Serum/plasma/CSF	Platelet release (serum), Val66Met genotype, physical activity	Inconsistent in serum/plasma; CSF more consistent but invasive	Not feasible (invasive/lab-based)	Outcome measure only	★
Lactate/lactylation	Blood/NIRS/CSF	Physical exertion, metabolic state	Mechanistic interest; lactylation is emerging hypothesis; no clinical validation	Emerging (NIRS for lactate; lactylation not real time)	Research/future direction	☆

Abbreviations: PLV, phase-locking value; HRV, heart rate variability; RMSSD, root mean square of successive differences; LF/HF, low-frequency to high-frequency ratio; HEP, heartbeat-evoked potential; SNR, signal-to-noise ratio; BDNF, brain-derived neurotrophic factor; NIRS, near-infrared spectroscopy; CSF, cerebrospinal fluid. Evidence Level key: ★★ = strong correlational or mechanistic evidence, but closed-loop validation incomplete; ★ = preliminary or indirect evidence, currently exploratory; ☆ = speculative/emerging, no direct evidence available.

## 3. Real-Time Biomarkers for Brain Fatigue: From Electrophysiology to Molecular Signatures

The sensing module is the entry point of any closed-loop system; its performance determines the quality of all subsequent operations. An ideal biomarker for closed-loop sensing should change reliably with fatigue state, be detectable in real time, be minimally invasive, and show acceptable inter-individual consistency. In practice, however, no single biomarker satisfies all these criteria simultaneously—EEG offers excellent temporal resolution and portability but provides limited specificity regarding the underlying drivers of fatigue [146,147]; molecular biomarkers offer mechanistic insight but are currently difficult to measure in real time; and peripheral physiological signals provide complementary information about autonomic state but are subject to numerous confounds [148]. This section reviews three categories of biomarkers, from the most mature to the most emerging, and discusses how they might be integrated to provide a comprehensive, multi-scale picture of the fatigue state. The phenotypic distinctions outlined in Table 1 are reflected in the biomarker profiles discussed below.

### 3.1. EEG-Derived Biomarkers

Electroencephalography (EEG) is the most widely used sensing modality in current closed-loop systems due to its excellent temporal resolution, portability, and relatively low cost [149,150]. Several EEG features have been extensively investigated as fatigue markers and validated in both laboratory and real-world settings [151]. Spectral power ratios are among the most reliable features. The ratio of beta power to the sum of alpha, theta, and delta powers—often termed the Focus Index—provides a composite measure of alertness that has been validated in real-time cognitive state monitoring. This ratio captures the shift from low-frequency activity associated with drowsiness and disengagement (theta and delta) to high-frequency activity associated with active cognitive processing (beta) [152], and it has been shown to track fatigue development across sustained task performance in both laboratory and operational settings.

The beta/theta ratio, which reflects the balance between high-frequency and low-frequency activity, has shown consistent associations with fatigue in occupational settings [153], though its specificity for clinical fatigue remains to be established. This ratio has been shown to decline progressively during sustained cognitive tasks, with the rate of decline correlating with subjective fatigue ratings and objective performance decrements [153]. Importantly, the beta/theta ratio appears to have some degree of cross-individual generalizability, making it one of the more promising EEG features for closed-loop applications [154,155]. Frontal alpha asymmetry—calculated as the difference in alpha power between left and right frontal electrodes—has been proposed as a potential marker of motivational state, with preliminary evidence linking it to fatigue-related reductions in effort investment. Left frontal alpha power increases with reduced approach motivation [156], and several studies have found that greater left frontal alpha (indicating reduced left frontal activity) correlates with higher fatigue ratings and lower willingness to exert effort [157]. This feature may be particularly relevant to the motivational aspects of fatigue, as opposed to the purely cognitive or somatic aspects.

Beyond spectral features, functional connectivity metrics have revealed that fatigue is associated with widespread reorganization of large-scale brain networks [158,159]. Phase-locking value (PLV), which quantifies the consistency of phase relationships between EEG signals across different brain regions, has been shown to decrease in some fatigue studies, indicating reduced inter-regional communication and network integration, although findings are not entirely consistent across study populations [160,161]. Graph-theoretic measures—including global efficiency (the average inverse shortest path length across the network) and clustering coefficient (a measure of local network segregation)—have suggested that fatigue may be associated with reduced global efficiency and increased clustering in some studies, suggesting that the brain network becomes more “modular” and less integrated during fatigue [162,163]. This may reflect a functional reorganization in which the brain shifts from a globally integrated mode—optimal for complex, demanding tasks—to a more locally segregated mode that is energetically less costly but also less flexible [163]. Cross-frequency coupling, particularly theta–gamma phase-amplitude coupling, has been shown to predict fatigue-related changes in information processing, with reduced coupling between the phase of theta oscillations (thought to reflect cognitive control) and the amplitude of gamma oscillations (thought to reflect local neuronal computation) correlating with performance decrements [164,165,166].

A critical point to emphasize is that EEG features reflect the “network-level output” of brain fatigue—they are the electrophysiological manifestations of underlying molecular events—rather than the primary drivers themselves. The changes in EEG spectral power and connectivity that accompany fatigue are ultimately caused by changes in neurotransmitter levels, metabolic state, and neuroinflammatory activity that are not directly visible in the EEG signal. This is both an advantage and a limitation: EEG provides an integrated, real-time measure of network function that reflects the combined effect of all underlying molecular processes; however, it does not provide information about which molecular processes are responsible for the observed changes, which may limit the ability of EEG-guided systems to deliver biologically specific interventions.

Despite their widespread use, EEG-derived features face three fundamental limitations. First, lack of specificity: spectral power changes are similarly affected by sleep deprivation, task difficulty, emotional arousal, circadian phase, and caffeine. Second, temporal inertia: EEG changes evolve over minutes rather than seconds, challenging “real-time” triggering. Third, clinical validation gap: nearly all EEG fatigue studies have been conducted in laboratory settings with healthy individuals; generalization to clinical populations remains unproven. EEG features should therefore be considered part of a multimodal sensing strategy, not a standalone validated trigger.

### 3.2. Peripheral Physiological Biomarkers

Peripheral physiological signals offer complementary information to EEG, particularly regarding autonomic nervous system function and the brain–body interactions that are increasingly recognized as central to fatigue. Heart rate variability (HRV), derived from electrocardiogram or photoplethysmography, reflects parasympathetic and sympathetic balance and has been shown to change during sustained cognitive tasks in laboratory settings [167], though its utility for ambulatory fatigue monitoring is constrained by multiple confounders discussed below. The ratio of low-frequency to high-frequency HRV power, which reflects sympathovagal balance, has been shown to increase during sustained cognitive tasks, indicating a shift toward sympathetic dominance. Time-domain measures of HRV, such as the root mean square of successive differences (RMSSD), which primarily reflects parasympathetic activity, have been reported to decrease during sustained task performance [168,169], but these changes are not specific to fatigue. The direction of these changes is generally consistent across studies, suggesting that autonomic nervous system function is systematically altered in fatigue states, with reduced parasympathetic activity and increased sympathetic drive [170].

However, the relationship between HRV and fatigue is complex and context-dependent [171]. Different HRV metrics appear to capture different aspects of autonomic adaptation: time-domain measures may reflect the rapid, phasic components of autonomic response to changing task demands, while frequency-domain measures may reflect more tonic, sustained autonomic states [172,173]. Moreover, the relationship between HRV and fatigue may be moderated by individual differences in baseline autonomic function, which vary widely across individuals and are influenced by age, fitness, and genetic factors [174]. Nonetheless, the combination of central (EEG) and peripheral (HRV) measures has been adopted in several closed-loop systems and appears to enhance classification accuracy compared to either modality alone, presumably because the two modalities capture complementary aspects of the fatigue state—EEG reflecting central neural dynamics and HRV reflecting autonomic outflow and stress responses [174,175].

Heartbeat-evoked potentials (HEPs), which represent the cortical response to cardiac signals as captured by the EEG, provide a direct measure of interoceptive processing—the brain’s representation of internal body states [176,177]. Given that brain fatigue is increasingly understood as involving altered interoception—the perception and interpretation of bodily signals—HEPs represent a promising but underexplored biomarker for closed-loop sensing [178,179,180]. The amplitude of the HEP has been shown to correlate with trait interoceptive accuracy, and several studies have found reduced HEP amplitude in conditions characterized by fatigue, suggesting altered cortical processing of cardiac signals in the fatigued state [181]. The advantage of HEPs is that they can be derived from the same EEG recording that provides the spectral and connectivity features described above, making them an “add-on” biomarker that requires no additional hardware—only appropriate signal processing to extract the cardiac-locked cortical responses [182,183]. The integration of HEPs into EEG-based closed-loop systems could provide a measure of interoceptive state that is complementary to the more general measures of cortical arousal and network connectivity provided by spectral and connectivity features.

HRV-based sensing is vulnerable to confounders: physical activity [184], respiratory rate [185], emotional arousal [185], medication (beta-blockers, anticholinergics) [185], caffeine [186], sleep quality [187], age, and fitness [185]. Reliable frequency-domain HRV metrics require 5 min recording windows, introducing temporal inertia that is at odds with real-time control. Time-domain measures like RMSSD can be computed over shorter windows but primarily reflect parasympathetic activity and are also respiration-sensitive [188,189]. These limitations suggest HRV is better suited as a slow baseline or contextual variable than a primary real-time trigger.

### 3.3. Molecular Biomarkers

While EEG and HRV detect the immediate, fast-changing correlates of fatigue, molecular biomarkers provide insight into the underlying biological processes that drive fatigue persistence and recurrence [23,190]. These molecular signals operate on slower timescales—minutes to hours to days—and therefore provide information about the “baseline” state of the individual, the trajectory of fatigue development, and the potential drivers of fatigue that may not be apparent from electrophysiological signals alone [191]. The integration of molecular biomarkers into closed-loop systems represents a crucial frontier that could transform the specificity and effectiveness of fatigue interventions. This section examines four classes of molecular biomarkers and proposes a framework for their integration into closed-loop architectures.

#### 3.3.1. Inflammatory Cytokines

Peripheral cytokines inform systemic inflammatory status [139,192], but they are not direct readouts of central neuroinflammation [140]. Among the most extensively studied molecular markers are the pro-inflammatory cytokines, particularly IL-6, TNF-α, and IL-1β [141,193,194]. Elevated circulating levels of these cytokines have been consistently observed in fatigue-predominant conditions, including ME/CFS, post-COVID syndrome, and cancer-related fatigue [142]. In a large cohort of post-COVID patients, elevated IL-6 levels at six months post-infection were associated with persistent fatigue, and in ME/CFS patients, circulating levels of IL-6 have been shown to correlate with subjective fatigue severity [142,195]. These associations have been replicated across multiple studies, though effect sizes vary considerably across populations. However, the relationship between peripheral and central cytokine levels is not straightforward—cytokines are large molecules that do not readily cross the intact blood–brain barrier, and peripheral levels may not fully reflect central neuroinflammation [196,197]. The central levels of cytokines, as measured in cerebrospinal fluid, show stronger correlations with fatigue severity but are not feasible for real-time monitoring [198]. Nevertheless, peripheral cytokine levels can serve as “slow variables” that inform baseline fatigue susceptibility and reflect the overall inflammatory burden that an individual carries, even if they cannot be used for millisecond-scale decision-making [199,200]. The practical utility of inflammatory markers for closed-loop systems may therefore lie in their use as “state estimators” that set the individual’s baseline inflammatory status and adjust trigger thresholds for the faster EEG and HRV signals.

#### 3.3.2. HPA-Axis Markers

The hypothalamic–pituitary–adrenal (HPA) axis, which coordinates the body’s stress response, shows characteristic alterations in fatigue states [35,201]. The most consistently observed pattern is hypocortisolism—reduced morning cortisol levels and a flattened diurnal slope—which has been reported in ME/CFS, fibromyalgia, and burnout syndrome [202]. This pattern is thought to reflect chronic HPA axis suppression following prolonged stress, and it may contribute to fatigue through its effects on energy regulation, sleep–wake cycles, and immune function [203]. Cortisol can be measured in saliva, which is a more practical medium for repeated monitoring, and salivary cortisol measurements have been shown to correlate reasonably well with serum cortisol levels [204,205]. The diurnal pattern of cortisol secretion—which shows characteristic time-dependent changes across the day—could be used by closed-loop systems to calibrate trigger thresholds based on the individual’s circadian phase, a possibility that we explore further in the Future Directions Section.

#### 3.3.3. Brain-Derived Neurotrophic Factor (BDNF)

Brain-derived neurotrophic factor (BDNF) is a key regulator of synaptic plasticity [206,207], neuronal survival, and long-term potentiation [208]. However, evidence for BDNF as a fatigue biomarker must be interpreted with attention to the measurement compartment. Serum BDNF—the most commonly reported measure—has shown inconsistent associations with fatigue, with some studies reporting reduced levels in fatigue-predominant conditions while others find no significant difference [209]. Plasma BDNF is less frequently measured and shows even weaker correlations. Cerebrospinal fluid (CSF) BDNF is more directly relevant to central nervous system function, but is rarely measured due to the invasiveness of lumbar puncture. Importantly, serum and plasma BDNF do not necessarily reflect brain BDNF dynamics, as the majority of circulating BDNF is stored in platelets and released upon activation, and its relationship to brain BDNF concentration is indirect at best [210,211]. Serum/plasma BDNF may therefore be useful as a peripheral outcome measure of systemic neurotrophic status, but it is not feasible for real-time sensing and should not be interpreted as a direct measure of brain BDNF availability. The BDNF Val66Met polymorphism, which affects activity-dependent BDNF secretion, is an important moderating factor contributing to inter-individual variability in neuroplasticity and therapeutic response [212,213].

#### 3.3.4. Metabolic Markers

ATP depletion and adenosine accumulation represent a fourth class of molecular markers that directly reflect the cellular energy state. While ATP and adenosine are not currently measurable in real time using non-invasive methods, lactate levels can be monitored using microdialysis or near-infrared spectroscopy, and there is growing interest in developing wearable biosensors for metabolites [214,215]. Lactate is of particular interest not only as a marker of metabolic stress but also as a signaling molecule in its own right, with roles in synaptic plasticity, neuroprotection, and, via histone lactylation, epigenetic regulation of inflammatory genes [125,216]. The potential to monitor lactate or other metabolic markers in real time would represent a significant advance in closed-loop sensing, as metabolic signals would provide a direct window into the energy state of the brain that is currently inaccessible with EEG or HRV alone.

#### 3.3.5. A Framework for Integration

The integration of molecular biomarkers into closed-loop systems requires careful consideration of their temporal dynamics [217]. Unlike EEG features, which change on a second-to-second basis, molecular markers evolve over minutes to hours or even days. We propose a “fast-slow variable” framework in which EEG and HRV serve as fast variables that guide immediate intervention decisions, while molecular biomarkers serve as slow variables that establish baseline fatigue susceptibility, set individual-specific trigger thresholds, and provide information about the underlying biological trajectory. In this framework, the molecular variables are not used for the fast, moment-to-moment decisions about intervention timing—these are made by the fast variables—but rather for the slower, more strategic decisions about how to parameterize the system for a given individual at a given time. For instance, an individual with high baseline inflammatory markers might have a lower threshold for intervention (i.e., the system would intervene earlier and more aggressively) than an individual with low inflammatory markers, and the threshold could be adjusted over time as the individual’s inflammatory state changes in response to intervention [218,219].

Biosensor technologies are advancing rapidly and may eventually enable the continuous monitoring of some molecular markers, though significant challenges remain [220]. Sweat-based electrochemical sensors for cortisol and inflammatory markers, microneedle arrays for interstitial fluid sampling, and tear-based biosensors for metabolic analytes are all in various stages of development [220,221]. When such sensors reach sufficient maturity, they will allow molecular biomarkers to move from “slow variables” that are measured periodically to “medium-speed variables” that can be monitored more continuously [222], potentially enabling a richer, multi-scale model of the individual’s fatigue state that incorporates information from electrophysiological, autonomic, and molecular domains simultaneously.

While these technological advances are encouraging, a more fundamental constraint must also be acknowledged: real-time sensing of molecular markers directly within the central nervous system is not currently feasible in humans. The molecular signals that can be measured peripherally—whether in blood, sweat, or saliva—are informative for baseline stratification but do not provide direct, continuous readouts of central neuroinflammatory or metabolic states. Thus, within the fast–slow variable framework, “slow variables” are, for the foreseeable future, constrained to periodic peripheral sampling rather than truly real-time central monitoring. Their role is therefore best understood as baseline calibration and threshold adjustment, complementing the faster electrophysiological signals rather than competing with them for real-time control.

A distinction must be made between biomarkers suitable for real-time closed-loop control and those useful for long-term monitoring or baseline stratification. For a signal to drive instantaneous stimulation adaptation, it must be measurable continuously and change on a sub-second to second timescale. Currently, only neurophysiological signals—EEG spectral features and connectivity metrics—meet this criterion, albeit with limitations in specificity and clinical validation as discussed in Section 3.1. Peripheral physiological signals such as HRV, while measurable in real time, operate on slower timescales (seconds to minutes) and are best suited as contextual or slow variables rather than primary triggers. Molecular biomarkers—cytokines, cortisol, BDNF, metabolites—operate on timescales of minutes to days; they cannot drive rapid stimulation adjustments but remain valuable for individualizing baseline thresholds, tracking treatment response, and informing long-term dosing decisions. This functional distinction is reflected in Table 3, where each biomarker is classified by its recommended role in closed-loop architecture.

The translational readiness of each biomarker discussed in Section 3 is summarized in Table 3.

## 4. Triggering Logics: From Reactive Detection to Predictive Intervention

The decision-making algorithm in closed-loop systems determines when intervention occurs based on biomarker data, unlike open-loop systems with fixed schedules. This section examines the evolution of triggering logic from simple thresholding to predictive control, showing a shift from state sensing to trajectory prediction for effective fatigue intervention.

### 4.1. Static Threshold Triggering

The simplest decision rule is threshold-based triggering: when a biomarker signal exceeds (or falls below) a predefined threshold, the system activates an intervention [223]. This approach is computationally simple, interpretable, and widely used in existing closed-loop systems for conditions like epilepsy, where seizure detection relies on thresholding of EEG amplitude or frequency features [224,225]. In the fatigue context, threshold triggering might be implemented by, for example, delivering transcranial stimulation whenever the beta/theta ratio drops below a certain value or whenever HRV decreases by more than a specified percentage from a resting baseline.

To illustrate the implementation of threshold-based triggering in practice, consider the closed-loop system developed for treatment-resistant post-traumatic stress disorder [226]. In this system, the threshold was defined as a significant elevation in amygdala low-frequency bandpower (specifically, a 2-standard-deviation increase from the individual’s baseline) measured from intracranial electrodes. When the signal exceeded this threshold, the system delivered a brief electrical pulse to the amygdala [227]. The threshold was derived from the individual’s own baseline recordings, recognizing that absolute power values vary substantially across individuals [228]. In the fatigue domain, an analogous approach might set the beta/theta ratio threshold at 70% of the individual’s pre-task baseline, with stimulation initiated when the ratio declines below this level. While this individual-referenced approach improves upon a fixed absolute threshold, it still requires the individual to have a stable baseline that can be measured before fatigue develops—a condition that may not hold in clinical settings where patients are already fatigued at the time of system initialization.

The appeal of threshold triggering lies in its parsimony and real-time applicability—it requires minimal computation, which is advantageous for low-power, wearable devices, and it can be implemented without sophisticated machine learning or individualized calibration beyond baseline determination. However, this approach suffers from several fundamental limitations that make it ill-suited to the complexity of brain fatigue. First, the threshold is static, whereas the relationship between biomarker values and fatigue state can vary across individuals (a value that indicates fatigue in one person may be within the normal range for another) [229], across time within the same individual (the same value may indicate fatigue at one point in the day but not at another due to circadian variations) [229,230], and across contexts (fatigue in a resting state may manifest differently from fatigue during active task performance). A threshold that is set correctly for one person at one time will inevitably be inappropriate for others or for the same person at other times [231].

Second, threshold-based systems are inherently reactive: they respond to a state that has already occurred, inevitably introducing a delay between fatigue onset and intervention [232,233,234]. Even if the threshold is calibrated to detect fatigue early—before subjective awareness—it is still responding to a state that has crossed a boundary, rather than anticipating the state before it has developed. This reactive nature is particularly problematic for fatigue, where the optimal intervention window may lie before the full development of symptoms rather than after they have become manifest.

Third, threshold triggering does not account for the trajectory of biomarker change [235,236]. A slow, gradual drift in a biomarker over time may be more informative about impending fatigue than a sudden fluctuation that crosses the threshold briefly, yet both would be treated equivalently [237]. For instance, a gradual decline in the beta/theta ratio over 30 min may predict fatigue onset more reliably than a sudden dip that occurs in response to a transient attentional lapse, but a threshold-based system would trigger on the sudden dip but not on the gradual decline, potentially missing the more clinically significant pattern. These limitations highlight the need for more sophisticated decision-making logics that can account for both individual differences and temporal context [238].

### 4.2. Pattern Recognition-Based Triggering

To overcome the limitations of static thresholding, more sophisticated systems employ machine learning classifiers that recognize fatigue states based on the overall pattern of multiple biomarker signals. Rather than relying on a single feature crossing a single threshold, these systems integrate information across channels, modalities, and time points to produce a holistic assessment of brain state [239,240].

The key advantage of pattern recognition approaches is their ability to extract features from multidimensional data in a way that captures synergistic relationships that might be missed by univariate thresholding. Multimodal feature fusion—combining EEG spectral features, connectivity measures, HRV parameters, and potentially molecular marker inputs—has been shown to enhance classification accuracy compared to single-modality approaches [241]. The decision boundary in such systems is learned from training data rather than being specified a priori, allowing for more flexible and context-sensitive triggering [242]. Machine learning classifiers—including support vector machines, random forests, and gradient boosting machines—have been successfully applied to distinguish fatigued from non-fatigued states [243,244].

However, pattern recognition systems, like threshold-based systems, are fundamentally reactive—they classify the current state based on present features without explicitly modeling future trajectories [245]. They can accurately identify that the individual is currently fatigued, but they cannot predict whether the individual will become fatigued in the next 5, 10, or 30 min [246]. This limits their utility for pre-emptive intervention because they cannot act until fatigue has already developed. Furthermore, pattern recognition systems require labeled training data, which is labor-intensive to collect and may not generalize across tasks or populations [247,248]. A classifier trained on one type of fatigue—say, fatigue from a sustained attention task—may not perform well on fatigue from a different type of task, such as a memory task, and a classifier trained on one population may not generalize to others [249]. These limitations have motivated the development of a third generation of triggering logics that go beyond classification of current state to prediction of future state.

### 4.3. Predictive Triggering

The most recent and conceptually radical evolution in triggering logics is the shift from reactive detection to predictive triggering. The core insight is that the optimal therapeutic window for brain fatigue interventions lies before, not after, the onset of subjective symptoms—once an individual consciously experiences fatigue, the opportunity for early intervention has already passed, and the system can at best restore function rather than prevent decline. Predictive triggering aims to forecast fatigue onset by modeling the trajectory of biomarkers over time, allowing the system to intervene proactively rather than reactively. This requires a shift from classification—learning the mapping between features and current labels—to sequence prediction—learning the mapping between the recent history of features and the future state of the system [250]. This is a more challenging problem that requires larger and more temporally structured datasets, but the potential benefits are substantial [251].

The slower dynamics of molecular biomarkers are particularly relevant for predictive triggering: changes in cytokine levels, cortisol patterns, or metabolic markers often precede the electrophysiological manifestations of fatigue by minutes to hours, making them potential early warning signals that could be incorporated into predictive algorithms. For example, a gradual rise in IL-6 over several hours might serve as a “slow-moving” signal that predicts an increased probability of fatigue onset in the coming hours [252,253], allowing the system to adjust trigger thresholds or initiate preparatory interventions. This temporal structure—with molecular changes preceding electrophysiological changes preceding subjective symptoms—provides a natural hierarchy of timescales that predictive algorithms can exploit.

**Figure 1:**
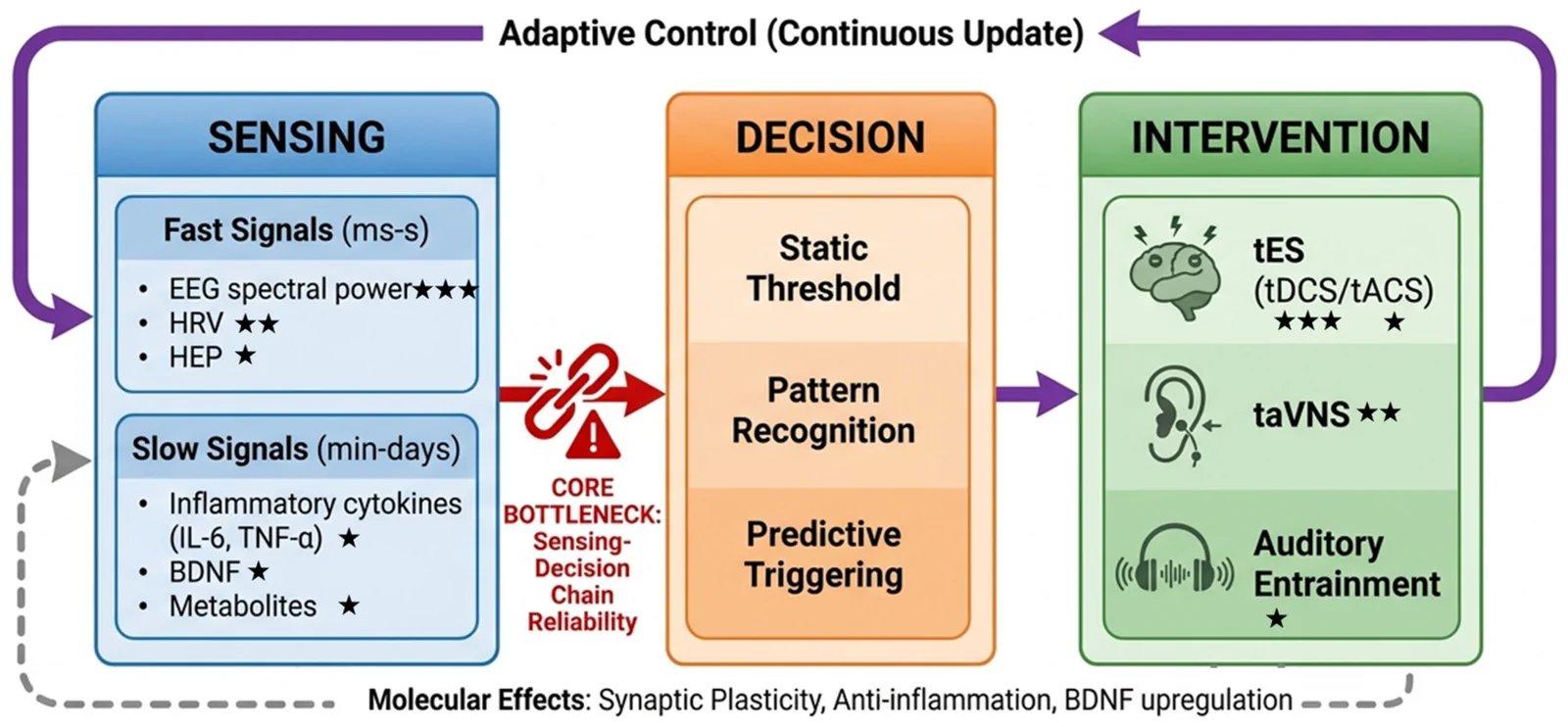
System architecture and core bottleneck of closed-loop neuromodulation for brain fatigue. The system comprises three core modules—Sensing, Decision, and Intervention. The Sensing module integrates fast signals (EEG features, HRV, HEP; milliseconds to seconds) for real-time state tracking and slow signals (inflammatory cytokines, BDNF, metabolites; minutes to days) for baseline calibration. The core bottleneck (red marker) lies between Sensing and Decision—the challenge of reliably translating multi-modal biological signals into individualized triggering decisions. The Decision module evolves from static threshold through pattern recognition to predictive triggering. The Intervention module includes tES (tDCS/tACS), taVNS, and auditory entrainment. Purple arrows indicate the adaptive closed-loop cycle with continuous parameter updates. Gray dashed arrows indicate post-intervention molecular effects—synaptic plasticity, anti-inflammation, and BDNF upregulation—which feed back to modulate subsequent sensing. The primary bottleneck of current systems is not stimulation technology but the reliability and individualization of the sensing–decision chain. PLV, phase-locking value; HRV, heart rate variability; HEP, heartbeat-evoked potential; BDNF, brain-derived neurotrophic factor; IL-6, interleukin-6; TNF-α, tumor necrosis factor-alpha; tES, transcranial electrical stimulation; tDCS, transcranial direct current stimulation; tACS, transcranial alternating current stimulation; taVNS, transcutaneous auricular vagus nerve stimulation. Markers indicate evidence readiness: ★★★ = validated in at least one closed-loop study; ★★ = strong evidence but closed-loop validation incomplete; ★ = preliminary/speculative.

The development of reliable predictive algorithms faces substantial hurdles, however. It requires longitudinal training data that captures the dynamics of fatigue across multiple timescales [254], which is difficult to collect outside of controlled laboratory settings. It also requires modeling individual-specific trajectories, as the relationship between biomarker dynamics and fatigue onset may vary substantially across individuals. And it carries the risk of false positives—predictions that trigger intervention when no fatigue would have occurred—which could lead to unnecessary or even disruptive interventions that undermine user trust and adherence [255,256]. Nonetheless, predictive triggering represents the logical culmination of the evolution toward truly adaptive closed-loop systems, and we consider it a key frontier for future research. The overall architecture of such a closed-loop system, from multi-scale sensing through decision-making to adaptive intervention—along with its core bottleneck—is illustrated in Figure 1.

The promise of predictive triggering depends, however, on careful attention to several methodological issues in model development and validation. Training such models requires longitudinal data with frequent, reliable ground-truth labels—currently scarce for clinical fatigue, as most available datasets are limited to laboratory-based occupational fatigue with session-level rather than continuous annotation. Cross-validation should be subject-level to prevent data leakage, and external validation on independent cohorts is essential before clinical deployment [257,258,259]. Class imbalance is also a practical concern: fatigue episodes are typically shorter than non-fatigue periods, and the cost of false positives (unnecessary stimulation, user annoyance, habituation risk) and false negatives (missed intervention, symptom worsening) must be weighed explicitly, with user adherence and safety as primary constraints [260,261]. Calibration drift—changes in biomarker baselines due to circadian rhythms, day-to-day variability, or disease progression—requires online recalibration mechanisms. Signal dropouts are inevitable in wearable use [138]; for prolonged gaps, a conservative default to “no stimulation” is prudent until signal quality is restored.

### 4.4. A Conceptual Algorithm for Fast–Slow Integration

The fast–slow variable framework, while conceptually appealing, requires operational specification. Below we present a conceptual algorithm to illustrate how fast variables (EEG/HRV, updated every 30–60 s) and slow variables (molecular markers, measured daily or per-session) might be integrated (Box 1). This is not a validated clinical algorithm; it is a template for future implementation and validation.

The specific numerical values for sampling intervals, window lengths, and adjustment increments would require empirical optimization in real-world deployment and are not prescribed here.

**Failure modes and handling (conceptual examples):**

The algorithm above is intended to illustrate a control logic, not a validated clinical protocol. In a practical implementation, several failure modes would need to be considered:

**False positives (stimulation when not needed):** A user-initiated override could be provided; if overrides occur frequently, the system might benefit from recalibration of the trigger threshold.

**False negatives (no stimulation when needed):** A user-triggered “on-demand” stimulation option could be considered; frequent reports of untreated fatigue might similarly inform threshold recalibration.

**Signal dropout (loss of EEG/HRV data):** The system should default to a conservative “no-stimulation” mode until signal quality is restored.

Importantly, the numerical adjustment values and decision rules illustrated above are purely illustrative and would require rigorous safety testing, mathematical stability analysis, and clinical validation before any implementation. Automatic threshold adjustments and patient-triggered stimulation carry risks of overstimulation, particularly in vulnerable populations, and must be evaluated in controlled settings with appropriate safety monitoring (see Section 7.4).

Box 1 **Pseudocode for fast–slow closed-loop control.**

// INITIALIZATION PHASE (conducted before first use, or daily at session start)

Measure slow variables:

    IL-6, TNF-α (serum/plasma): systemic inflammatory burden

    Cortisol diurnal profile (saliva, 3–4 time points): HPA axis status

    BDNF (serum/plasma, optional): neurotrophic baseline

Compute individual baseline for fast variables:

    EEG beta/theta ratio at rest (5 min, eyes-open)

    HRV RMSSD at rest (5 min)

Set trigger thresholds:

    beta/theta threshold = a reduced threshold relative to resting beta/theta ratio

        (illustrative value, not clinically validated)

    IF IL-6 > individual’s own baseline (or population reference median if baseline unavailable):

        threshold = threshold adjusted downward

    IF cortisol awakening response is blunted:

        threshold = threshold adjusted slightly downward

// ONLINE PHASE (continuous loop)

while (system is active):

    Compute EEG beta/theta ratio (moving average, short window)

    Compute HRV RMSSD (moving average, short window)

    Composite fatigue index F = (beta/theta_normalized + HRV_normalized)/2

    if (F < threshold for several consecutive windows):

        if (linear extrapolation predicts F will cross below threshold within a short predictive window):

            Deliver low-intensity stimulation (pre-emptive)

        else:

            Deliver standard-intensity stimulation

        Wait for a brief refractory period

    if (time since last slow update exceeds a defined interval, e.g., daily or per session):

        Re-measure slow variables; re-calibrate threshold

    if (user overrides occur repeatedly):

        adjust threshold upward

    if (user reports severe fatigue without prior trigger on multiple occasions):

        adjust threshold downward

## 5. Adaptive Intervention: Closed-Loop Neuromodulation Strategies and Molecular Mechanisms

A closed-loop system’s effectiveness depends on its intervention module, which delivers neural stimulation. This section distinguishes fixed-parameter from adaptive stimulation, reviews major closed-loop neuromodulation modalities for brain fatigue, and examines their molecular mechanisms. While Section 2 established fatigue’s molecular pathophysiology (disease mechanisms), Section 5.3 explores how neuromodulation interventions act on these molecular systems to produce therapeutic effects (treatment mechanisms).

### 5.1. From Fixed-Parameter to Adaptive Stimulation

A deceptively simple distinction separates true closed-loop systems from their open-loop counterparts: whether stimulation parameters are fixed or dynamically adjusted based on real-time feedback. Fixed-parameter closed-loop systems essentially operate as “detect-then-stimulate” devices—they trigger a predetermined stimulation protocol upon detecting a threshold event, but once triggered, the stimulation does not adapt to the brain’s changing state. These systems are an improvement over open-loop stimulation because they at least respond to the presence of a fatigue state, but they still do not account for the fact that the ideal stimulation parameters may change as the fatigue state evolves.

True adaptive systems, by contrast, implement a continuous “sense-decide-intervene-resense” cycle, in which stimulation parameters—including current intensity, pulse frequency, electrode configuration, and stimulation duration—are iteratively updated in response to ongoing biomarker signals [137]. This is fundamentally more challenging than fixed-parameter triggering because it requires an algorithm that can determine not only when to stimulate, but also how to stimulate—and to adjust that “how” in real time as the brain’s state evolves [44,262].

The theoretical advantage of adaptive stimulation is substantial: because fatigue fluctuates dynamically, a stimulation protocol that is appropriate at one time point may be suboptimal or even counterproductive at another [263]. For instance, a high-intensity stimulation that effectively counteracts early fatigue might become overstimulating as the individual approaches recovery, potentially causing adverse effects such as headache, discomfort, or disrupted sleep [143]. Conversely, a stimulation that is insufficiently intense for an individual in a state of deep fatigue might fail to have any therapeutic effect at all. Adaptive algorithms are designed to prevent these mismatches by continuously recalibrating to maintain the system in a state of dynamic equilibrium, providing just enough stimulation to counteract fatigue without producing side effects or disrupting the individual’s normal cognitive functioning [144].

The development of adaptive algorithms faces two fundamental challenges [145,264]. First, the temporal relationship between biomarker changes and the effects of stimulation is not instantaneous: there is a lag between intervention and response, which must be modeled to avoid overcorrection. If the algorithm adjusts stimulation too quickly based on a signal that reflects the system’s state before the previous intervention has taken effect, it may create oscillations or instability. If it adjusts too slowly, it may fail to match the dynamic trajectory of fatigue [265]. Second, the optimal stimulation parameters are highly individual—what works for one person may not work for another—requiring not only real-time adjustment but also person-specific initialization [266]. Approaches that combine pre-training on population data with online fine-tuning using individual feedback have been proposed, drawing from frameworks originally developed in adaptive deep brain stimulation for Parkinson’s disease.

### 5.2. Modalities of Closed-Loop Neuromodulation

A range of neuromodulation modalities has been adapted for closed-loop applications. We focus on three non-invasive modalities—tDCS, tACS, and taVNS—because they meet four criteria: non-invasiveness (permitting repeated use without surgical risk), portability (enabling ambulatory and home-based deployment), existing evidence of efficacy or mechanistic relevance to fatigue-related pathways, and closed-loop compatibility (all three can be integrated with EEG or physiological sensing in principle, though technical challenges remain) [267]. We also discuss auditory entrainment as an emerging adjunctive modality [268,269]. Invasive techniques (e.g., deep brain stimulation) and modalities with higher logistical demands (e.g., repetitive transcranial magnetic stimulation) are excluded because their risk profiles, cost, and infrastructure requirements currently preclude widespread use in fatigue populations [270]. Transcranial electrical stimulation (tES), including transcranial direct current stimulation (tDCS) and transcranial alternating current stimulation (tACS), is the most extensively investigated for brain fatigue due to its noninvasiveness, portability, and safety. tDCS modulates cortical excitability by altering resting membrane potentials—anodal stimulation increases excitability while cathodal stimulation decreases it. tACS entrains endogenous oscillatory activity to an external frequency for rhythm-specific network modulation. Both modalities have been successfully implemented in closed-loop configurations.

Non-invasive vagus nerve stimulation (taVNS) offers a complementary approach, targeting the autonomic nervous system through the auricular branch of the vagus nerve. Unlike tES, which directly modulates cortical networks, taVNS operates through brainstem pathways that project diffusely to widespread cortical and subcortical regions. The clinical rationale for taVNS in fatigue is twofold: it activates the cholinergic anti-inflammatory pathway, which may counteract neuroinflammation, and it modulates noradrenergic and serotonergic systems, directly addressing the neurotransmitter imbalances described earlier. Closed-loop taVNS systems have been proposed in which stimulation parameters are dynamically generated in response to EEG features extracted by convolutional and recurrent neural networks.

Auditory entrainment, delivered through bone-conduction audio, represents a newer and less invasive modality that leverages the powerful effects of rhythmic auditory stimulation on brain oscillations. The rationale for auditory entrainment in fatigue is that rhythmic auditory stimulation can entrain theta and alpha oscillations, potentially enhancing alertness and cognitive performance. This mechanism is thought to operate through the synchronization of neuronal populations to the external rhythm, which may improve the efficiency of information processing and reduce the energy cost of maintaining cognitive performance. Closed-loop systems that combine EEG sensing with adaptive audio stimulation have been developed, with stimulation parameters—frequency, intensity, and rhythm—dynamically adjusted based on real-time EEG measures of arousal and attentional state.

The key parameters, targets, and clinical evidence for the modalities discussed above are summarized in Table 4.

### 5.3. Molecular Correlates of Neuromodulation

The therapeutic effects of closed-loop neuromodulation are thought to be mediated by molecular changes at the synaptic, cellular, and systems levels, though direct evidence is still accumulating. Understanding these molecular correlates—how stimulation translates into lasting biological change—is essential not only for mechanistic interpretation but also for the rational optimization of stimulation parameters and the prediction of individual responses. This section examines the molecular mechanisms underlying the three major neuromodulation modalities, complementing the disease-focused molecular discussion in Section 2 by focusing on treatment mechanisms rather than disease mechanisms.

#### 5.3.1. Synaptic Plasticity and Neurotransmitter Modulation

At the synaptic level, tDCS and tACS modulate NMDA receptor-dependent synaptic plasticity—the same molecular machinery underlying long-term potentiation (LTP) and long-term depression (LTD) [131,274]. Anodal tDCS has been shown to increase the concentration of glutamate in stimulated cortical regions while reducing GABA, shifting the excitatory–inhibitory balance toward enhanced excitation [132,133]. These effects are NMDA receptor-dependent, as evidenced by the blockade of tDCS after-effects by NMDA receptor antagonists, and they are thought to be mediated by changes in the activity of calcium-calmodulin-dependent protein kinase II (CaMKII) and the insertion of AMPA receptors into the postsynaptic membrane [134,135,136]. The molecular time course of these effects—with early changes in receptor phosphorylation and trafficking giving way to longer-lasting changes in gene expression and protein synthesis—corresponds to the known time course of tDCS after-effects, which can persist for hours to days after a single stimulation session.

tACS, through rhythmic entrainment, may engage spike-timing-dependent plasticity, a Hebbian learning rule in which the precise timing of pre-synaptic and post-synaptic activity determines the direction and magnitude of synaptic change. By imposing an external rhythm on neuronal activity, tACS can create a consistent temporal relationship between pre-synaptic and post-synaptic firing patterns, thereby promoting synaptic strengthening or weakening according to the rules of spike-timing-dependent plasticity. This mechanism may be particularly relevant to the effects of tACS on network dynamics and cognitive performance, as it suggests that tACS can produce not just transient changes in neuronal activity but also lasting modifications of synaptic strength and network connectivity [275,276,277].

#### 5.3.2. Anti-Inflammatory Effects

The anti-inflammatory effects of neuromodulation are increasingly recognized as a key pathway relevant to fatigue [278]. Vagus nerve stimulation activates the cholinergic anti-inflammatory pathway, in which efferent vagal signals release acetylcholine that binds to nicotinic receptors on macrophages, inhibiting the release of pro-inflammatory cytokines—including TNF-α, IL-6, and IL-1β—through the NF-κB pathway [271,272]. This mechanism has been demonstrated in multiple animal models and in human studies, where vagus nerve stimulation has been shown to reduce circulating inflammatory markers [273]. Non-invasive brain stimulation may similarly modulate microglial activation and cytokine expression, though the mechanisms are less well characterized. The effect is thought to involve both direct effects on cortical regions that project to autonomic centers and indirect effects through the modulation of stress pathways and the HPA axis [279]. Given the central role of neuroinflammation in fatigue pathophysiology, these anti-inflammatory actions provide a molecular rationale for the use of neuromodulation in fatigue-predominant conditions that complements the more commonly discussed effects on synaptic plasticity and neurotransmitter balance [280].

#### 5.3.3. Neurotrophic Factors

BDNF, the most abundant neurotrophin in the brain, is consistently upregulated by various neuromodulation modalities [281]. BDNF promotes neuronal survival, synaptic growth, and LTP, and its upregulation may represent a common downstream pathway through which different neuromodulation techniques exert their beneficial effects [282]. Human studies have shown that a single session of tDCS increases serum BDNF levels, and repetitive stimulation may produce cumulative effects on neuroplasticity [283]. The mechanism is thought to involve activity-dependent BDNF secretion, which depends on the influx of calcium through voltage-gated calcium channels and the subsequent activation of intracellular signaling cascades—including the MAPK/ERK pathway and the PI3K/Akt pathway—that promote BDNF transcription and release. Individual differences in BDNF function—particularly the BDNF Val66Met polymorphism, which reduces activity-dependent BDNF secretion—contribute to inter-individual variability in neuroplasticity and therapeutic response [284,285]. This may explain why some individuals show robust improvements in fatigue following neuromodulation while others show little or no response.

#### 5.3.4. Molecular Imaging Approaches

Molecular imaging techniques—particularly PET and magnetic resonance spectroscopy (MRS)—are crucial tools for assessing these molecular effects in vivo [286,287]. PET can measure neuroinflammation through TSPO ligands, which bind to activated microglia and provide a measure of central inflammatory state [288]. MRS can measure neurotransmitter concentrations, including glutamate, GABA, and the ratio of these excitatory to inhibitory transmitters, as well as metabolites such as lactate and N-acetyl-aspartate (NAA), which is a marker of neuronal integrity [289]. These techniques have been used in human studies to demonstrate that neuromodulation can produce measurable changes in TSPO binding (indicating reduced neuroinflammation), glutamate/GABA balance, and NAA levels (indicating improved neuronal health) [290]. The integration of molecular imaging into closed-loop system design remains a distant prospect, as these techniques require large, expensive equipment and cannot be performed in real time. However, they can provide crucial information for the design of stimulation protocols and the interpretation of clinical outcomes, as well as for identifying biomarkers that might predict individual responses to treatment [291].

A design constraint worth making explicit is the timescale mismatch between the immediate electrical effects of stimulation and its downstream molecular consequences. When tDCS, tACS, or taVNS is applied, the direct effect is electrical—changes in membrane potential and firing rate—occurring within milliseconds to seconds. By contrast, the molecular changes that are thought to mediate longer-term therapeutic benefits, including BDNF upregulation [292], cytokine reduction [293], and synaptic protein synthesis [294], unfold over hours to days [295]. This dissociation has practical implications for closed-loop architecture [296]. From a control perspective, this suggests the value of distinguishing between a faster loop (seconds–minutes), in which EEG/HRV could inform immediate state modulation, and a slower loop (days–weeks), in which molecular markers might guide cumulative stimulation parameters [297,298]. An algorithm that attempts to use fast EEG signals to drive slow molecular outcomes would be operating across mismatched timescales; conversely, waiting for molecular shifts before adjusting stimulation would likely miss the rapid fluctuations of fatigue [299]. Future system design may therefore benefit from explicitly considering these two timescales as separate but interacting control layers.

## 6. Multimodal Integration: Enhancing Robustness Through Synergy

The complexity of brain fatigue, arising from the interaction of multiple molecular pathways and neural networks, naturally suggests that single-modality approaches may be insufficient for reliable sensing and effective intervention. Multimodal integration—combining multiple sensing modalities and/or multiple intervention techniques—has consequently emerged as a key trend in closed-loop system design, aiming to enhance robustness and efficacy through synergistic effects that are not achievable with any single modality alone.

The theoretical necessity for multimodal integration follows from the information-theoretic concept of incompleteness: any single sensing modality provides only a partial view of the fatigue state [300,301]. EEG provides information about cortical dynamics but is blind to subcortical activity, autonomic state, and molecular drivers [302]. HRV provides information about autonomic function but is influenced by numerous confounds including physical activity, respiratory patterns, and emotional state [303,304]. Molecular biomarkers provide information about underlying biological processes but are not currently available in real time [222]. Each modality thus captures a different aspect of the fatigue state, and each has its own blind spots and limitations. The combination of multiple modalities can, in principle, provide a more complete picture that is robust to the limitations of any individual modality [151]. This is the same principle that underlies multimodal integration in clinical diagnosis—combining clinical history, physical examination, laboratory tests, and imaging—and it is just as relevant to the automated diagnosis of fatigue states [305]. Formally, if we consider the true fatigue state as a high-dimensional vector in a state space, each sensing modality provides a projection of that vector onto a lower-dimensional subspace. No single projection is guaranteed to capture all the information needed for accurate state estimation, but the combination of multiple projections can approximate the full state vector with greater fidelity [306]. This is the information-theoretic foundation for multimodal integration [307].

At the sensing level, the combination of EEG and HRV is the most established multimodal strategy [308,309]. EEG provides direct information about cortical dynamics—the oscillations, connectivity patterns, and network states that reflect central neural function—while HRV reflects autonomic outflow and its regulation by the brainstem and higher centers [310,311]. Together, they offer a more comprehensive picture of the brain–body interaction than either modality alone [312]. EEG-HRV fusion has been shown to enhance classification accuracy in fatigue detection compared to either modality alone, with improvements in both sensitivity and specificity [313]. The BUZZ-PR system exemplifies this approach, using both EEG spectral features and HRV parameters as inputs to a machine learning classifier.

Emerging work suggests that the addition of molecular sensing—through sweat-based biosensors, microneedle arrays, or other novel technologies—could further enhance sensing robustness by capturing the “slow variables” discussed earlier [314,315]. This would represent a further expansion of the multimodal sensing concept, from the current “central plus peripheral” model to a “central plus peripheral plus molecular” model that captures information at three timescales (milliseconds for EEG, seconds for HRV, and minutes to hours for molecular markers) and three levels of biological organization (neural, autonomic, and molecular). The practical implementation of such multi-level sensing remains a significant challenge, but the potential benefits—a more robust and biologically grounded assessment of fatigue state—are substantial [316,317].

At the intervention level, multimodal stimulation strategies have the potential to target multiple fatigue-relevant pathways simultaneously [318]. The integration of tDCS (direct neuromodulation of cortical excitability) with bone-conduction audio (indirect entrainment through auditory pathways) is one example of how multiple intervention mechanisms can be combined within a single device. The rationale for such combinations is that different modalities may have different time courses of action—with tDCS producing relatively slow changes in cortical excitability and audio entrainment producing more immediate changes in brain oscillations—and combining them may produce both immediate and sustained benefits. Other multimodal interventions include the combination of transcranial stimulation with cognitive training, which may enhance the effects of both modalities by providing concurrent cognitive engagement and neuromodulation [319,320]. The combination of neuromodulation with physiological interventions like exercise represents another potential multimodal strategy, though it is less amenable to closed-loop implementation due to the logistical constraints of exercise administration [321].

The synergy between multimodal sensing and multimodal intervention is where the full potential of closed-loop architecture becomes apparent. Systems that sense at multiple levels and intervene through multiple modalities could theoretically implement sophisticated control strategies, such as switching from one intervention modality to another based on the pattern of detected biomarkers, or combining modalities to achieve additive or synergistic effects. Despite this promise, multimodal integration faces significant technical and practical challenges. Signal interference between simultaneous stimulation and sensing modalities—particularly the contamination of EEG by tES currents—remains a fundamental obstacle to continuous “sense-while-stimulate” operation. Most current systems operate in a “stimulate-pause-sense” alternation pattern, which sacrifices continuity. Computational demands increase substantially with each additional modality, posing challenges for wearable, battery-powered devices that must operate under strict power and size constraints. Finally, user acceptance may decrease with device weight, complexity, and cost—all of which tend to increase with multimodality. Overcoming these hurdles is essential to realize the full potential of multimodal closed-loop systems that can dynamically sense and intervene across multiple biological levels to effectively manage the multifaceted nature of brain fatigue.

Simultaneous sensing and stimulation—mentioned above as one of the multimodal integration challenges—deserves additional technical consideration. For tES, the electrical current applied through scalp electrodes produces large artifacts in concurrently recorded EEG, often saturating amplifiers or introducing stimulation-frequency harmonics that obscure underlying neural signals [322,323]. The most common workaround is a “stimulate–pause–sense” alternation pattern, in which stimulation is briefly interrupted every few minutes to acquire a clean EEG sample. While feasible, this approach sacrifices the continuity of closed-loop control [324]. For taVNS, the artifact issue is less severe, but vagal stimulation can alter HRV, confounding autonomic signals that share overlapping physiological pathways [325,326]. Auditory entrainment presents minimal artifacts, making it the most compatible with simultaneous sensing—but its weaker therapeutic effects limit its standalone utility [327]. Hardware-level innovations, such as active artifact cancellation and interleaved sensing, are needed before continuous sense-while-stimulate becomes practical for tES-based systems [328]. These considerations reinforce the broader point that multimodal integration, while conceptually attractive, must contend with real-world technical constraints beyond algorithmic design.

## 7. Translational Challenges

The translation of closed-loop neuromodulation from laboratory to real-world applications requires overcoming three fundamental challenges: causality, individual variability, and engineering constraints.

### 7.1. The Causality Problem

Are detected biomarkers causes, consequences, or mere correlates of fatigue? The answer determines the validity of using them as control signals. If a biomarker is merely a consequence, intervening based on it may have no effect on the fatigue state itself—like adjusting the speedometer rather than the engine.

The problem is acute at the molecular level. Elevated inflammatory cytokines are consistently observed in fatigue conditions, but the direction of causality is difficult to establish [329,330]. The evidence suggests bidirectional causality—inflammation contributes to fatigue, and fatigue (through reduced activity and disrupted sleep) perpetuates inflammation. This circularity makes it unclear whether targeting inflammation will reduce fatigue or merely treat a secondary manifestation [252,331]. Similarly, in post-COVID syndrome, it remains debated whether neurological symptoms arise from direct viral effects, cerebrovascular disorders, or systemic inflammation—each implying a different intervention strategy [332,333].

Temporal relationships can help resolve causality: if molecular changes precede fatigue onset, they are more likely causal or predictive; if they follow, they are consequences. However, establishing temporal precedence requires longitudinal data collection with frequent sampling, which is challenging in practice [334]. Definitive resolution requires integration across multiple lines of evidence—observational, experimental (intervention studies targeting specific pathways), and predictive modeling—in a way rarely achieved in any single study [335].

### 7.2. Inter-Individual Variability

Individual differences are a pervasive challenge. Anatomical variations affect tES current distribution; baseline EEG characteristics differ dramatically across individuals; fatigue susceptibility is influenced by genetics, lifestyle, and comorbidities [336,337].

Molecular-level differences are particularly relevant. The BDNF Val66Met polymorphism affects activity-dependent BDNF secretion and tDCS response. Baseline inflammatory status shifts the threshold at which neuroinflammation contributes to fatigue [338,339]. Genetic variation in neurotransmitter systems influences susceptibility and treatment response [340]. These differences mean that a biomarker value indicating fatigue in one person may be normal for another.

Individualisation—establishing individual-specific baselines, thresholds, and algorithm parameters—has become a central principle [341]. However, it requires pre-intervention data collection, which may be impractical in real-world settings, and increases system complexity [342]. Whether individualisation should apply to all components or be restricted to critical parameters (e.g., trigger thresholds) remains an open question, depending on application, population, and available data.

### 7.3. Engineering Constraints: The Information–Energy–Time Trilemma

Translation imposes a fundamental trilemma among information quality (signal fidelity), energy efficiency (battery life), and time responsiveness (latency). In laboratory settings all three are accommodated; in wearable devices, optimising any one dimension typically comes at the expense of the others.

#### 7.3.1. Signal Quality

Dry/semi-dry electrodes are more practical than wet electrodes but have higher impedance and motion artifact susceptibility, degrading signal-to-noise ratio [343,344]. Novel electrode materials and artifact correction algorithms are in development but consume computational resources—adding to the energy burden.

#### 7.3.2. Computational Efficiency and Latency

Real-time operation imposes strict latency requirements: intervention decisions must be made within seconds or milliseconds to be clinically meaningful [345]. This limits the complexity of deployable algorithms [346]. Edge computing is essential for low latency and data privacy but adds hardware and power demands—a direct trade-off between algorithmic sophistication and battery life.

#### 7.3.3. Power Consumption

Continuous EEG, real-time processing, and intermittent stimulation all consume power [347]. Achieving full-day operation requires careful system-level optimisation: low-power analog front-ends, efficient DSP, and power-efficient stimulation circuits. Energy harvesting technologies are not yet mature for the power levels required [348].

#### 7.3.4. Systems-Level Perspective

There is also the challenge of system-level dysfunction that cannot be addressed by engineering alone. Glymphatic system dysregulation in ME/CFS may contribute to brain fog [349]; a daytime fatigue system might be ineffective if the underlying problem is impaired nocturnal waste clearance. Post-exertional malaise requires predicting and preventing symptom worsening [350]. These system-level interactions between sleep, circadian rhythms, and fatigue pose conceptual challenges extending beyond the device itself [351].

### 7.4. Safety, Tolerability, and the Challenge of Post-Exertional Malaise

The translation of closed-loop neuromodulation into clinical practice—particularly for vulnerable populations such as ME/CFS and Long COVID—requires explicit consideration of safety, tolerability, and the unique risk of post-exertional malaise.

#### 7.4.1. Risk Profiles

The three main modalities discussed in this review have distinct safety profiles. tDCS and tACS are generally well-tolerated, with the most common adverse effects being mild skin irritation, tingling, or headache [352]; serious adverse events such as seizure are rare at conventional parameters but remain a contraindication in individuals with epilepsy or implanted electronic devices [353]. taVNS may cause local discomfort, dizziness, or nausea in some users, with vasovagal syncope reported in rare cases [354]; it is contraindicated in individuals with known vagal or carotid sinus hypersensitivity. Auditory entrainment carries minimal risk, though it may cause auditory fatigue or annoyance in some users, and is contraindicated only in severe hearing impairment.

#### 7.4.2. Post-Exertional Malaise (PEM)

A special consideration for ME/CFS and Long COVID populations is PEM—a delayed (typically 24–72 h post-exertion) exacerbation of fatigue, flu-like symptoms, cognitive dysfunction, and pain [355]. Importantly, a transient improvement in cognitive performance or energy during or immediately after neuromodulation does not guarantee safety; it may be followed by a PEM-related worsening that outweighs any acute benefit. This concern is not hypothetical: in clinical practice, exercise or cognitive therapy that pushes patients beyond their energy envelope has been associated with PEM exacerbation [356], and a similar risk may apply to neuromodulation that temporarily enhances function without addressing underlying energy deficits.

PEM risk is not well captured by standard safety reporting in neuromodulation trials, as most studies exclude ME/CFS and Long COVID populations or do not systematically monitor delayed symptom exacerbation. For closed-loop systems intended for use in these populations, we suggest that future clinical protocols incorporate: (i) gradual dose titration rather than fixed-intensity protocols; (ii) systematic monitoring of fatigue and symptom status in the 48–72 h following stimulation sessions; and (iii) explicit stopping rules for symptom exacerbation. Patient education is also critical: users should be informed that transient improvement during stimulation does not necessarily indicate overall benefit, and that any delayed symptom worsening should be reported even if the acute stimulation period felt beneficial.

#### 7.4.3. Long-Term Use

The effects of repeated daily stimulation over months or years are unknown for fatigue populations [357]. Potential risks include tolerance/habituation and homeostatic plasticity [358]. While tDCS and taVNS are considered safe for short-term use, data beyond 30 sessions are scarce [359]. We suggest that closed-loop systems for chronic fatigue incorporate periodic washout breaks and longitudinal monitoring of fatigue severity, mood, sleep quality, and adverse events, with formal stopping criteria for any significant deterioration. These considerations are precautionary and highlight the need for systematic safety monitoring in future long-term studies.

## 8. Future Directions

Several frontier directions have the potential to transform closed-loop neuromodulation from an emerging concept into a practical tool. Beyond the modalities discussed above, emerging methods—including low-intensity focused ultrasound, optogenetics-inspired approaches (where translational), and closed-loop electromagnetic stimulation—could offer improved spatial resolution, deeper targeting, or complementary mechanisms. While many remain at preclinical or early clinical stages, their potential for fatigue applications warrants closer investigation.

### 8.1. Predictive Architectures

The shift from reactive to predictive control requires forecasting fatigue trajectories using longitudinal data and sequence prediction algorithms [360]. The goal is to transform closed-loop systems from “fatigue responders” to “fatigue preventers.” Molecular biomarkers, with slower dynamics (changes hours before EEG manifestations), could be particularly valuable as early signals.

### 8.2. Real-Time Molecular Sensing

The “fast-slow variable” framework points toward integrating molecular biomarkers into real-time control. Sweat-based electrochemical sensors, microneedle arrays, and tear-based biosensors are advancing rapidly [361,362]. When mature, they will augment rather than replace electrophysiological sensing, providing a multi-scale picture of biological state.

### 8.3. Chronobiological Principles

Fatigue has strong temporal structure—diurnal rhythms, ultradian cycles [363]. Incorporating chronobiology (cortisol awakening response, circadian regulation) could anticipate natural fluctuations in fatigue susceptibility. The DyNeuMo-2c system, adjusting parameters based on circadian phase, provides a precedent.

### 8.4. BCI–Neuromodulation Integration

Brain–computer interfaces and closed-loop neuromodulation are converging [364,365]. Future systems may infer cognitive state, task demands, and performance trajectory, adjusting parameters accordingly—beyond current “detect-and-respond” architectures [366].

### 8.5. Long-Term Efficacy and Tolerability

Effects of repeated daily stimulation over months or years are unknown. Risks include tolerance/habituation and adverse effects. Understanding molecular mechanisms—receptor downregulation, altered homeostatic plasticity—is essential for developing safe, effective long-term interventions [367,368].

## 9. Conclusions

This critical review has examined the evidence and conceptual framework for closed-loop neuromodulation in brain fatigue, with a focus on integrating electrophysiological and molecular biomarkers into the sensing–decision–intervention architecture. The fast–slow variable framework offers a principled approach to accommodating fatigue’s multiple timescales, while the molecular pathophysiology provides biological rationale for biomarker selection. However, several critical gaps must be acknowledged: no fatigue-specific closed-loop system has been clinically validated, real-time central molecular sensing does not exist, predictive algorithms remain largely conceptual, and long-term safety data are absent. Despite these gaps, the review articulates the sensing–decision bottleneck as the primary constraint and offers a practical template for fast–slow architecture with explicit operational parameters. Translation will require validated algorithms, safety protocols for vulnerable populations, and user-centered design. The technical components exist in prototype form; what remains is their rigorous integration into safe, effective systems. Realizing this vision will require parallel progress in hardware (spatial targeting, dose precision, modality versatility) and in individualized sensing–decision algorithms. Both remain areas of active development, and neither can be neglected in the translation of closed-loop neuromodulation to clinical practice.

## Figures and Tables

**Table 1:** Phenotypic classification of brain fatigue: implications for closed-loop neuromodulation.

Fatigue Phenotype	Primary Clinical Context	Core Measurement	Temporal Scale	Relevance to Closed-Loop
Central/neurological fatigue	Multiple sclerosis, Parkinson’s disease, stroke, TBI	FSS, MFIS, cognitive batteries, EEG	Hours to days (persistent, fluctuating)	High—primary target; EEG/HRV biomarkers show consistent changes
Post-infectious fatigue	Long COVID, ME/CFS, post-EBV	Symptom diaries, PEM provocation, actigraphy	Days to weeks, with acute exacerbations	Medium-high—slow variables (cytokines, cortisol) especially relevant; caution with PEM
Cancer-related fatigue	Post-chemo/radiotherapy, advanced malignancy	Brief Fatigue Inventory, FACIT-F	Weeks to months (persistent, slow-changing)	Medium—more chronic; suited for outcome monitoring rather than real-time triggering
Occupational/cognitive fatigue	Healthy individuals during sustained tasks (driving, air traffic control, intensive cognitive work)	EEG (beta/theta ratio, alpha power), PVT, subjective VAS	Minutes to hours (task-dependent, reversible with rest)	High for prevention in healthy populations; strongest EEG validation exists here, but not a “disease” target
Fatigability	Aging, deconditioning, early neurodegeneration	Performance decrement over standardized task (e.g., grip strength, walking speed)	Minutes (within-task decline)	Low—primarily physical; overlaps with but is distinct from brain fatigue
Sleepiness/somnolence (EXCLUDED)	Sleep deprivation, narcolepsy, sleep apnea	MSLT, EEG theta/delta activity, Epworth Sleepiness Scale	Minutes to hours	Distinct from brain fatigue—explicitly excluded from this review

FSS, Fatigue Severity Scale; MFIS, Modified Fatigue Impact Scale; TBI, traumatic brain injury; EBV, Epstein–Barr virus; PEM, post-exertional malaise; FACIT-F, Functional Assessment of Chronic Illness Therapy–Fatigue; PVT, psychomotor vigilance task; VAS, visual analogue scale; MSLT, multiple sleep latency test.

**Table 4:** Stimulation parameters and clinical applications in fatigue.

Modality	Typical Parameters	Target	Session Protocol	Evidence in Fatigue	Rationale
tDCS	1–2 mA, 20–30 min	DLPFC (cognitive), M1 (motor)	1–2/day, 5–10 days	MS: FSS reduced ~1.5 points in RCTs [38,39,40]; cancer-related: mixed	Most studied; portable; EEG-compatible
tACS	0.5–2 mA, 5–40 Hz, 20–30 min	Frontal/parietal (frequency-specific)	1/day, 5–10 days	Cognitive fatigue in healthy (alpha-tACS enhances alertness); limited in clinical fatigue	Frequency-specific entrainment; can target endogenous rhythms
taVNS	1–3 mA, 20–30 Hz, 200–500 µs, 20–60 min	Auricular vagus (cymba conchae, tragus)	1–2/day, 2–4 weeks	ME/CFS, Long COVID: reduced fatigue severity in small trials [271,272,273]; fibromyalgia (preliminary)	Activates cholinergic anti-inflammatory pathway; targets autonomic–immune axis
Auditory entrainment	4–40 Hz rhythmic pulses, 70–80 dB, 15–60 min	Auditory pathways → cortical entrainment	As-needed or scheduled	Occupational fatigue (driving, sustained attention); minimal evidence in clinical fatigue	Minimal risk; low cost; easily combined with other modalities—adjunctive only

## Data Availability

No new data were created or analyzed in this study. Data sharing is not applicable to this article.

## Abbreviations

The following abbreviations are used in this manuscript:

BCI	Brain–Computer Interface
BDNF	Brain-Derived Neurotrophic Factor
CNS	Central Nervous System
DAMP	Damage-Associated Molecular Pattern
DLPFC	Dorsolateral Prefrontal Cortex
EBV	Epstein–Barr Virus
ECG	Electrocardiography
EEG	Electroencephalography
FACIT-F	Functional Assessment of Chronic Illness Therapy–Fatigue
FSS	Fatigue Severity Scale
GABA	Gamma-Aminobutyric Acid
HEP	Heartbeat-Evoked Potential
HPA	Hypothalamic–Pituitary–Adrenal
HRV	Heart Rate Variability
IDO	Indoleamine-2,3-Dioxygenase
IL-1β	Interleukin-1 Beta
IL-6	Interleukin-6
LTD	Long-Term Depression
LTP	Long-Term Potentiation
M1	Primary Motor Cortex
ME/CFS	Myalgic Encephalomyelitis/Chronic Fatigue Syndrome
MFIS	Modified Fatigue Impact Scale
MRS	Magnetic Resonance Spectroscopy
MSLT	Multiple Sleep Latency Test
NAA	N-Acetylaspartate
NF-κB	Nuclear Factor Kappa-B
NIRS	Near-Infrared Spectroscopy
NMDA	N-Methyl-D-Aspartate
PEM	Post-Exertional Malaise
PET	Positron Emission Tomography
PPG	Photoplethysmography
PVT	Psychomotor Vigilance Task
RMSSD	Root Mean Square of Successive Differences
ROS	Reactive Oxygen Species
SNR	Signal-to-Noise Ratio
tACS	Transcranial Alternating Current Stimulation
taVNS	Transcutaneous Auricular Vagus Nerve Stimulation
TBI	Traumatic Brain Injury
tDCS	Transcranial Direct Current Stimulation
tES	Transcranial Electrical Stimulation
TNF-α	Tumor Necrosis Factor-Alpha
TSPO	Translocator Protein
VAS	Visual Analogue Scale
